# Primary CNS Lymphoma Presenting With Seizures in a Virologically Suppressed HIV Patient With Near‐Normal CD4: A Case Report From Uganda

**DOI:** 10.1002/ccr3.72447

**Published:** 2026-03-29

**Authors:** Abdisalam Ahmed Sandeyl, Abdisamad Guled Hersi, David Elia Saria, Farah Dubad Abdi, Venance Emmanuel Mswelo, Sura Daniel Elias, Elias Amare Hailu, Hailemariam Kassahun Bekele

**Affiliations:** ^1^ Internal Medicine Department Kampala International University, Western Campus Ishaka Uganda; ^2^ Neurosurgery Department Kampala International University, Western Campus Ishaka Uganda; ^3^ Oncology Department Kampala International University, Western Campus Ishaka Uganda

**Keywords:** antiretroviral therapy, facial palsy, HIV infections, magnetic resonance imaging, primary central nervous system lymphoma, seizures

## Abstract

We report a case of primary central nervous system lymphoma (PCNSL) in a 40‐year‐old man with well‐controlled HIV infection, virologic suppression, and a near‐normal CD4 count who presented with new‐onset generalized seizures and left lower motor neuron facial palsy. Brain MRI showed a large left temporal lobe lesion with irregular ring enhancement, marked vasogenic edema and mild midline shift. Lumbar puncture was deferred due to mass effect. The patient was stabilized with anticonvulsants and supportive care and transferred for stereotactic biopsy and oncologic management. Stereotactic brain biopsy confirmed PCNSL, and the patient was initiated on high‐dose methotrexate‐based chemotherapy with adjunctive dexamethasone. This case highlights that PCNSL can occur despite immunologic preservation in HIV and may present acutely with seizures. Early contrast‐enhanced MRI, careful timing of corticosteroid use relative to biopsy, and timely referral are essential, particularly in resource‐limited settings.

## Key Clinical Message

Primary CNS lymphoma may occur even in virologically suppressed HIV patients with near‐normal CD4 counts and may initially present with seizures or focal neurological deficits. Early contrast‐enhanced MRI and timely referral for biopsy are essential to avoid diagnostic delay, especially in resource‐limited settings.

## Graphical abstract

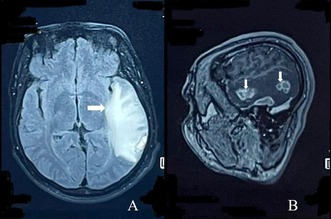

**Abbreviations**
ART: antiretroviral therapy
CD4: cluster of differentiation 4 (a type of T‐cell)
EBV: Epstein–Barr virus
FLAIR: fluid‐attenuated inversion recovery
HIV: human immunodeficiency virus
MRI: magnetic resonance imaging
PCNSL: primary central nervous system lymphoma
PLHIV: people living with HIV
TB LAM:
*Mycobacterium tuberculosis* lipoarabinomannan
TLD: Tenofovir–Lamivudine–Dolutegravir

## 1 Introduction

Primary central nervous system lymphoma (PCNSL) is a rare, aggressive B‐cell non‐Hodgkin lymphoma restricted to the brain, spinal cord, meninges, or eyes [1]. Historically, PCNSL in people living with HIV (PLHIV) has been associated with severe immunosuppression and Epstein–Barr virus (EBV)‐driven lymphomagenesis [2]. Typical symptoms include focal deficits, seizures, headaches, and cognitive changes [3].

MRI is the cornerstone of diagnosis; PCNSL commonly appears as homogeneously enhancing or ring‐enhancing lesions with marked vasogenic edema [4]. In PLHIV, the differential diagnosis includes toxoplasmosis, tuberculoma, and other opportunistic infections [5]. Biopsy remains the diagnostic gold standard, though in resource‐limited settings, clinical and radiologic features often inform initial diagnostic consideration when biopsy is delayed or unavailable [6].

With expanding ART coverage, PCNSL is increasingly reported in patients with higher CD4 counts and virologic suppression, posing diagnostic challenges because clinicians may not initially suspect lymphoma in immunologically stable individuals [7].

We present a case of PCNSL in a virologically suppressed HIV patient with a near‐normal CD4 count, highlighting diagnostic difficulties in a resource‐limited setting.

## 2 Case Presentation

### 2.1 Case History and Examination

A 40‐year‐old man living with HIV for 7 years, maintained on Tenofovir–Lamivudine–Dolutegravir (TLD) with good adherence, developed new‐onset generalized tonic–clonic seizures lasting approximately 5 min, accompanied by loss of consciousness, tongue biting, and urinary incontinence, and presented to the Emergency Department of Kampala International University Teaching Hospital on the same day. Afterward, he experienced a persistent diffuse headache but denied fever, trauma, limb weakness, sensory changes, or systemic symptoms. He had no history of CNS opportunistic infections.

He was hemodynamically stable but mildly confused, with a Glasgow Coma Scale score of 14/15 (E4 V4 M6). Motor strength, tone, reflexes, sensation, and coordination were intact. A left lower motor neuron facial palsy was evident, manifested by incomplete eye closure, loss of forehead wrinkling, and difficulty puffing the cheek. Fundoscopy was normal, and there were no meningeal signs.

### 2.2 Investigations and Differential Diagnosis

Laboratory tests showed a CD4 count of 535 cells/μL (reference range: 500–1500 cells/μL) and an HIV viral load of 76 copies/mL, confirming good immunologic and virologic control. Toxoplasma gondii IgG/IgM antibodies and urine TB LAM ( *Mycobacterium tuberculosis* lipoarabinomannan) were negative. Complete blood count and electrolytes were normal.

**FIGURE 1:**
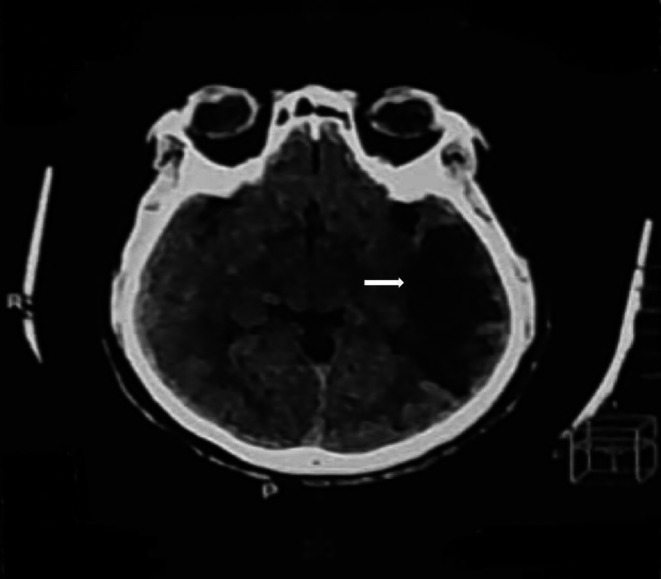
Brain CT of a 40‐year‐old virologically suppressed HIV patient with Primary CNS lymphoma. Axial non‐contrast brain CT image demonstrating a poorly defined hypodense lesion in the left temporal lobe with surrounding vasogenic edema and mild mass effect, without evidence of intracranial hemorrhage.

**FIGURE 2:**
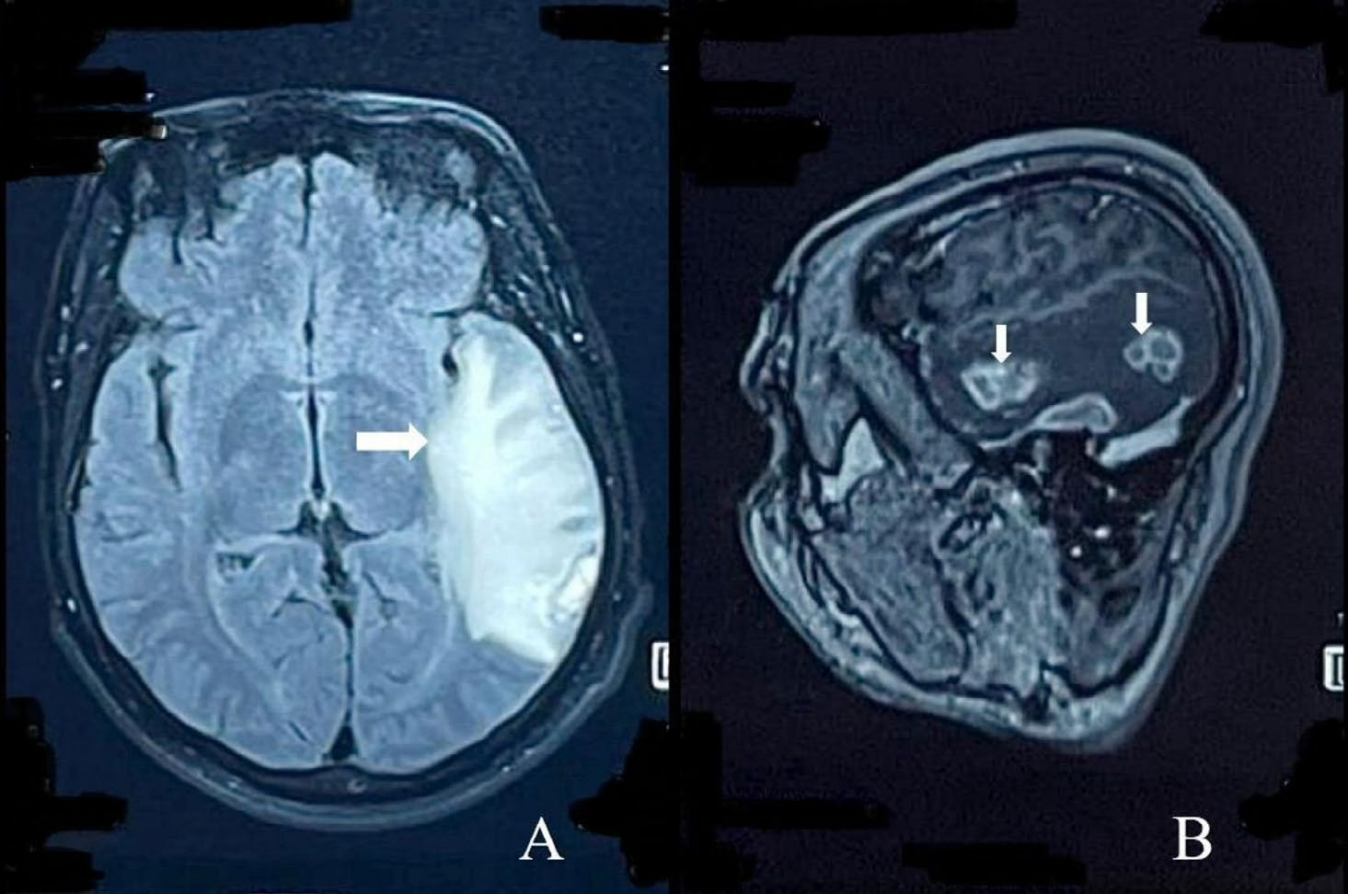
Brain MRI of a 40‐year‐old virologically suppressed HIV patient with Primary CNS lymphoma. (A) Axial T2/FLAIR image showing a large, ill‐defined hyperintense lesion in the left temporal lobe with vasogenic edema and mild compression of the left lateral ventricle. Subtle rightward midline shift is noted. (B) Sagittal post‐contrast T1‐weighted image demonstrating multifocal irregular ring‐enhancing lesions corresponding to the temporal lobe mass with marked vasogenic edema, highly suggestive of primary CNS lymphoma.

Initial non‐contrast brain CT performed on admission demonstrated a poorly defined hypodense lesion in the left temporal lobe with surrounding vasogenic edema and mild mass effect, without evidence of intracranial hemorrhage (Figure 1). This prompted further evaluation with contrast‐enhanced brain MRI within 48 h for better lesion characterization. MRI revealed a large, ill‐defined T2/FLAIR hyperintense lesion centered in the left temporal lobe with marked vasogenic edema, mild compression of the left lateral ventricle, and subtle rightward midline shift. Post‐contrast T1‐weighted images showed multifocal irregular ring‐enhancing lesions, radiologically highly suggestive of PCNSL (Figure 2A,B).

Differential diagnoses included toxoplasmosis, tuberculoma, and other space‐occupying lesions. A summary of the differential diagnoses, key clinical features, neuroimaging characteristics, and supporting/excluding arguments is presented in Table 1. Lumbar puncture was deferred due to mass effect and radiologic concern for raised intracranial pressure and herniation risk, based on contrast‐enhanced MRI findings; no other contraindications were identified at the time.

**TABLE 1:** Differential diagnoses in a virologically suppressed HIV patient with primary CNS lymphoma.

Differential diagnosis	Key clinical features	Neuroimaging characteristics	Arguments for	Arguments against/reasons excluded
Primary CNS lymphoma	Focal deficits, seizures, headache	Deep/temporal lesions; homogeneous or irregular ring enhancement with marked vasogenic edema	MRI features characteristic; lower motor neuron facial palsy; Biopsy‐confirmed	
Toxoplasmosis	Fever, headache, confusion; usually CD4 < 100	Multiple basal ganglia ring‐enhancing lesions	Common in HIV	Negative serology; high CD4; atypical imaging
Tuberculoma	Headache, fever, weight loss	Ring‐enhancing lesions with variable edema	TB prevalent in Uganda	Negative urine TB LAM test; no systemic TB symptoms
High‐grade glioma	Seizures, progressive focal deficits	Single irregular ring enhancement with mass effect and central necrosis	Can cause seizures	Patient age younger; imaging more suggestive of lymphoma
Brain abscess	Fever, rapid deterioration	Smooth ring enhancement with central diffusion restriction	Can cause seizures	No fever, normal labs, imaging not typical

*Note:* Key clinical features, neuroimaging characteristics, and supporting or excluding arguments for each differential diagnosis considered in a virologically suppressed HIV patient with primary CNS lymphoma.

Abbreviation: TB LAM, *Mycobacterium tuberculosis* lipoarabinomannan test.

### 2.3 Treatment and Outcome

The patient received intravenous phenytoin (1 g loading dose followed by 250 mg twice daily for 5 days) for seizure control. Intravenous paracetamol (1 g three times daily for 3 days, followed by as‐needed dosing) was administered for headache relief. Dexamethasone was initially withheld prior to biopsy to avoid potential reduction in diagnostic yield. Supportive care included seizure precautions and monitoring for raised intracranial pressure.

Stereotactic biopsy was unavailable at our facility; therefore, after stabilization, the patient was transferred within 5 days of admission to Mulago National Referral Hospital for neurosurgical biopsy and oncologic management. At Mulago National Referral Hospital, a stereotactic brain biopsy was performed. Histopathological examination revealed diffuse sheets of large atypical lymphoid cells with prominent nucleoli. Immunohistochemistry demonstrated strong positivity for CD20 and CD79a, while CD3 and CD10 were negative. The Ki‐67 proliferation index was approximately 80%. These findings were consistent with diffuse large B‐cell lymphoma, confirming the diagnosis of PCNSL.

Following histopathological confirmation of PCNSL, the patient was initiated on high‐dose methotrexate‐based chemotherapy (3.5 g/m^2^ intravenously, with planned cycles every 2 weeks) along with intravenous dexamethasone (8 mg three times daily for 5 days followed by gradual taper over 2 weeks). High‐dose methotrexate (HDMTX) is the cornerstone of therapy for PCNSL due to its ability to penetrate the blood–brain barrier, while corticosteroids help reduce peritumoral edema and improve neurological symptoms.

At one‐month follow‐up, he remained seizure‐free, had no new neurological deficits, and was receiving ongoing treatment under the oncology team.

## 3 Discussion

### 3.1 Atypical Presentation in Virologically Suppressed HIV

This case highlights an uncommon presentation of PCNSL in a patient with preserved CD4 immunity and virologic suppression. Though PCNSL classically occurs in advanced HIV, reports in immunologically stable individuals are increasingly recognized in the ART era [8], complicating clinical suspicion.

Although PCNSL typically presents subacutely over weeks to months with progressive neurological deficits, our patient developed acute‐onset generalized seizures over a single day, which is unusual [9, 10]. This presentation may be explained by the temporal lobe location and marked vasogenic edema, which can lower the seizure threshold. Such cases highlight the broad spectrum of neurological manifestations and underscore that PCNSL should remain in the differential even when onset appears sudden.

### 3.2 Differential Diagnosis and Neuroimaging

PCNSL remains challenging to differentiate from opportunistic CNS infections. Negative toxoplasmosis serology and urine TB LAM helped eliminate common mimics. The MRI features: ring enhancement, deep‐seated temporal involvement, and extensive vasogenic edema, are characteristic of PCNSL [11, 12]. The co‐occurrence of seizures and an isolated lower motor neuron facial palsy underscores the broad neurological spectrum of CNS lymphoma [13]. The differential diagnoses considered, along with supporting and opposing evidence, are summarized in Table 1. Lesion location and combined risk patterns have been shown to influence outcomes in PCNSL, highlighting the need for careful evaluation even in atypical presentations [14].

### 3.3 Management Considerations

The withholding of corticosteroids before biopsy is recommended because steroids may induce rapid tumor regression, reducing histopathological diagnostic yield [15]. Early seizure control, neuroimaging, and expedited referral for biopsy are essential components of management, especially in resource‐limited settings.

High‐dose methotrexate (HDMTX) remains the cornerstone of PCNSL induction therapy, with systematic reviews demonstrating favorable response rates and progression‐free survival when used in combination treatment protocols [16]. In this patient, HDMTX‐based chemotherapy was initiated shortly after biopsy confirmation, illustrating the importance of timely intervention.

### 3.4 Challenges in Resource‐Limited Settings

Limited access to MRI, lack of on‐site stereotactic biopsy, and delays in oncology referral remain major obstacles. Such constraints commonly delay diagnosis in Sub‐Saharan Africa [17]. Strengthening referral systems and diagnostic pathways is essential to improve timely management and patient outcomes.

### 3.5 Clinical Implications

Clinicians should remain alert to the possibility of PCNSL even in seemingly immunologically stable PLHIV who present with seizures or focal deficits. Early MRI, avoidance of premature corticosteroid use, and timely referral for biopsy are crucial for optimizing outcomes.

This case underscores that PCNSL can occur despite good immunologic control, highlighting the importance of maintaining high index of suspicion, prompt MRI, and timely oncologic evaluation in resource‐limited settings.

## Author Contributions

**Abdisalam Ahmed Sandeyl:** conceptualization, project administration, writing – original draft, writing – review and editing. **Abdisamad Guled Hersi:** conceptualization, resources. **David Elia Saria:** data curation, resources. **Farah Dubad Abdi:** investigation, writing – review and editing. **Venance Emmanuel Mswelo:** investigation, writing – review and editing. **Sura Daniel Elias:** investigation, resources. **Elias Amare Hailu:** investigation, resources. **Hailemariam Kassahun Bekele:** supervision, writing – review and editing.

## Funding

The authors have nothing to report.

## Consent

Written informed consent was obtained from the patient for publication of this case report and any accompanying images.

## Conflicts of Interest

The authors declare no conflicts of interest.

## Data Availability

All data supporting the findings of this case report are contained within the article. No additional datasets were generated or analyzed.
